# Nanotube Slidetronics

**DOI:** 10.1021/acs.jpclett.3c02681

**Published:** 2023-12-21

**Authors:** Wei Cao, Michael Urbakh, Oded Hod

**Affiliations:** Department of Physical Chemistry, School of Chemistry, The Raymond and Beverly Sackler Faculty of Exact Sciences and The Sackler Center for Computational Molecular and Materials Science, Tel Aviv University, Tel Aviv 6997801, Israel

## Abstract

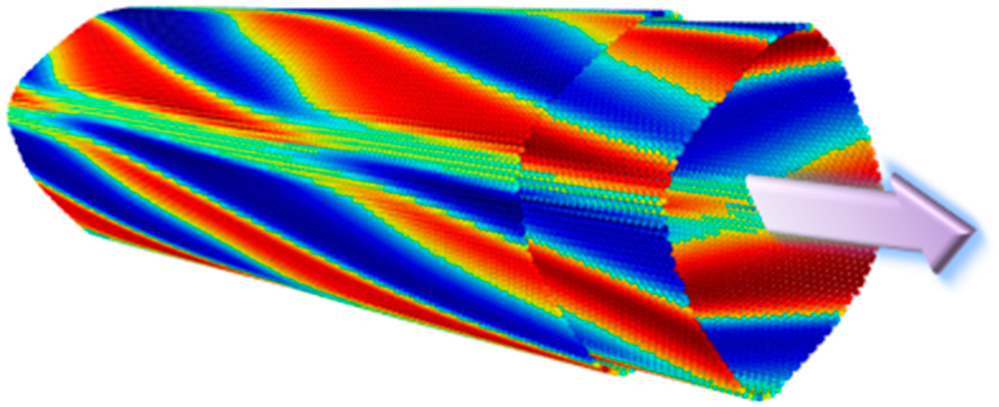

One-dimensional slidetronics is predicted for double-walled boron-nitride nanotubes. Local electrostatic polarization patterns along the body of the nanotube are found to be determined by the nature of the two nanotube walls, their relative configuration, and circumferential faceting modulation during coaxial interwall sliding. By careful choice of chiral indices, chiral polarization patterns can emerge that spiral around the nanotube circumference. The potential usage of the discovered slidetronic effect for low-dimensional nanogenerators is briefly discussed.

Ferroelectricity—a material state of spontaneous electric polarization that can be switched via external electric fields—serves as the basis for numerous practical applications.^1,2^ Traditionally, ferroelectricity has been considered as a bulk phenomenon, appearing in materials that possess a non-centrosymmetric unit-cell. Recently, interfacial ferroelectricity was discovered in two-dimensional (2D) material stacks that break inversion symmetry.^3^ This was first demonstrated for marginally twisted parallelly stacked *h*-BN bilayers exhibiting surface reconstruction with adjacent domains of opposite polarization. Reversible domain wall shifts, induced by external electric fields, resulted in domain polarization switching, leading to the emergence of the field of slidetronics.^4−6^ The interfacial localization of electric polarization in such constructs brings new opportunities to tune their ferroelectric properties. Specifically, multiple polarization states of cumulative nature can be designed and manipulated by controlling the multilayer stacking sequence.^7,8^ Furthermore, the plethora of available layered material building blocks provides a combinatorial playground for the construction of homogeneous^9−13^ and heterogeneous quasi-2D ferroelectric materials.^14−16^

Spontaneous electric polarization has also been predicted to emerge in quasi-one-dimensional (1D) layered material structures. In particular, single-walled carbon nanotubes have been suggested to exhibit intrinsic electric polarization perpendicular to their surface, due to curvature induced rehybridization (intermediate between sp^2^ and sp^3^) of the π orbitals that leads to charge redistribution.^17,18^ This effect can be further enhanced in single-walled boron-nitride nanotubes due to the polar nature of the B–N bond, leading to axial polarization^19^ and piezoelectric response.^20^ In multinanotube architectures, such as nanotube bundles or coaxial double-walled nanotubes, charge transfer between the coupled nanotubes can further influence the overall emergent polarization.^21−23^

Unlike their single-walled counterparts, multiwalled nanotubes often exhibit circumferential faceting, with achiral or chiral facet patterns, depending on the chiral angle difference between adjacent tube walls.^24−29^ The nature of these superstructures may strongly influence the electric polarization profile of the quasi-1D systems. Notably, by inducing interwall sliding, superstructure dynamics occurs exhibiting periodic unfaceting, refaceting, and facet rotations.^30^ The corresponding dynamical variations in the electric polarization maps manifest a novel realization of 1D-slidetronics.

To demonstrate this, we consider first the simple case of the achiral parallelly stacked^31^ zigzag (ZZ) (55,0)@(63,0) double-walled boron nitride nanotube (DWBNNT, Figure 1) with an outer wall diameter of *D* ≈ 50 Å and an interwall distance of 3.2 Å, close to the equilibrium *h*-BN planar bilayer separation of 3.35 Å.^32^ Here, the notation (*n*_1_,*m*_1_)@(*n*_2_,*m*_2_) represents an inner (*n*_1_,*m*_1_) tube wall coaxially aligned inside an outer (*n*_2_,*m*_2_) shell. We initially position the two tube walls in a parallel configuration (resembling the AB or BA stacking modes of the planar bilayer) such that they fully overlap; namely, they are not coaxially shifted with respect to each other. This initial structure is relaxed using a dedicated classical interlayer potential (ILP)^33,34^ yielding a faceted circumference (see Figure 1a and Methods) of 8-fold rotational symmetry. All facets are characterized by radially polar BA stacking domains, as depicted by the local polarization registry index (LPRI)^35^ map in Figure 1a (see Supporting Information (SI) section 1 for further details). To induce a slidetronic effect, the outer tube wall is shifted gradually in the axial direction with respect to the inner wall, and the system is allowed to fully relax after each shift step, while fixing the axial coordinate of all atoms (see SI section 2 for further information and the corresponding sliding energy profiles). The resulting facet variations are presented in Figure 1b–f, showing periodic facet rotations, with a period of 4.35 Å along the complete coaxial sliding cycle (see Figure 1a–f, SI section 2, and SI Movie S1) that corresponds to the hexagonal lattice vector along the armchair (AC) direction. These superstructure variations are clearly manifested in the LPRI maps, indicating a stacking mode modulation between the BA, intermediate, and AB configurations, accompanied by radial polarization switching, thus manifesting a pronounced slidetronic effect (Figure 1a–f and SI Movie S1).

**Figure 1:**
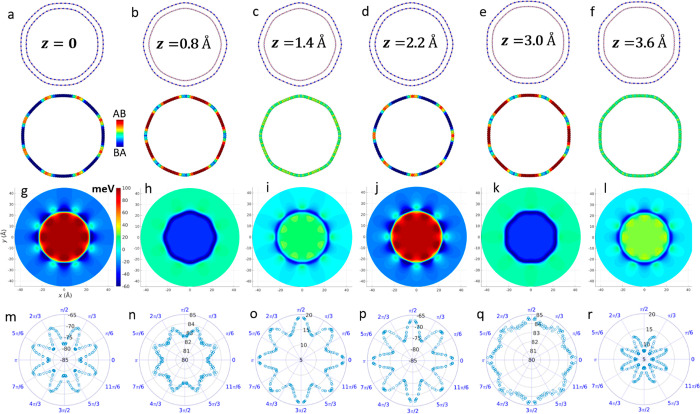
Axial interwall shift induced superstructure and radial polarization variations calculated for the ZZ (55,0)@(63,0) DWBNNT of preoptimized inner and outer wall diameters of 43.8 and 50.2 Å, respectively. (a–f) Cross-sectional views of the relaxed DWBNNT for several coaxial shifts, *z* = 0, 0.8, 1.4, 2.2, 3.0, and 3.6 Å. In each panel, the upper illustration represents the atomic structure and the lower illustration superimposes the corresponding LPRI map on the outer wall atomic positions (see color bar in panel a). (g–l) DFT calculated electrostatic potential difference maps (with respect to the same individual walls) plotted along the (001) face of the DWBNNTs presented in panels a–f, respectively. The color bar appearing in panel g is common to panels g–l. (m–r) Polar diagrams presenting the difference in electrostatic potential (in meV) calculated outside the tube at a radius of *r* = 35 Å and inside the tube at a radius of *r* = 5 Å. These values are obtained for each angle by radially averaging over a range of 0.02 Å and axially averaging over the length of the unit cell.

A quantitative analysis of the predicted polarization profile variations is provided by density functional theory (DFT) calculations (see Methods). Figure 1g–l presents the difference between the electrostatic potential DFT map of the DWBNNT and those of the corresponding individual walls across the (001) face. Clearly, both the angular and radial distributions of the electrostatic potential strongly depend on the DWBNNT superstructure, which is dictated by the difference in the number of circumferential unit-cells of the outer and inner tube walls and the interwall displacement.^29^ This is further reflected in the polar diagrams presented in Figures 1m–r, showing that the axially averaged radial polarization varies from being nearly isotropic to strongly anisotropic as a function of interwall displacement. Notably, despite the fact that the global polarization in the faceted DWBNNT nearly vanishes due to symmetry considerations, the predicted electrostatic variations, associated with internal charge density redistribution (see SI section 3.1), should be measurable via local experimental probing. Considering, for example, the azimuthal angle direction θ = π/2, the local radial potential energy differences vary periodically from −78 to 85 meV, with coaxial interwall sliding (see bottom panels of Figure 1).

We note that smaller radius ZZ DWBNNTs, e.g., the (30,0)@(38,0) system with an outer wall diameter of *D* ≈ 24 Å and an interwall distance of 3.2 Å, which do not exhibit faceting, show very small polarization values (∼6 meV, see SI section 3.2). Furthermore, antiparallelly stacked ZZ DWBNNTs demonstrate similar facet variations;^30^ however, due to the lack of AB or BA facet stacking configurations, they exhibit considerably smaller polarization values and azimuthal polarization anisotropy (see SI section 3.3).

Two other experimentally accessible observables are the DWBNNT bandgap and work function. For the polar parallelly stacked (55,0)@(63,0) DWBNNT we find coaxial displacement induced bandgap variations of ∼0.4 eV accompanied by work function variations of ∼0.1 eV (see SI section 3.4).

Similar features are found for parallelly stacked AC (31,31)@(36,36) DWBNNT (Figure 2) with an outer wall diameter of *D* = 49.7 Å and an interwall distance of 3.45 Å. Upon structural relaxation, the system forms five facets that vary periodically (with a period of 2.5 Å) along the complete coaxial sliding cycle (see Figure 2a–d, SI section 2, and SI Movie S2). Notably, while in the ZZ DWBNNT the AB or BA stacking configurations appear separately at the facet regions (Figure 1a–f), our LPRI analysis reveals that in the AC case they appear simultaneously. This is also reflected in the difference maps between the DFT electrostatic potential of the DWBNNT and those of the corresponding individual walls (Figure 2e–h). Due to the larger interlayer distance and the smaller spread of the polar stacking modes over the facet regions, lower variations of the electrostatic potential energy radial differences (−10 to 16 meV, see Figure 2i,k at the azimuthal angle direction θ = π/2) are observed during coaxial sliding (Figure 2i–l) compared to the ZZ DWBNNT of similar diameter considered above. This can be attributed to the fact that the interwall charge density redistribution in the ZZ DWBNNT case is considerably more delocalized than that of the corresponding AC DWBNNT of similar diameter (see SI section 4.1).

**Figure 2:**
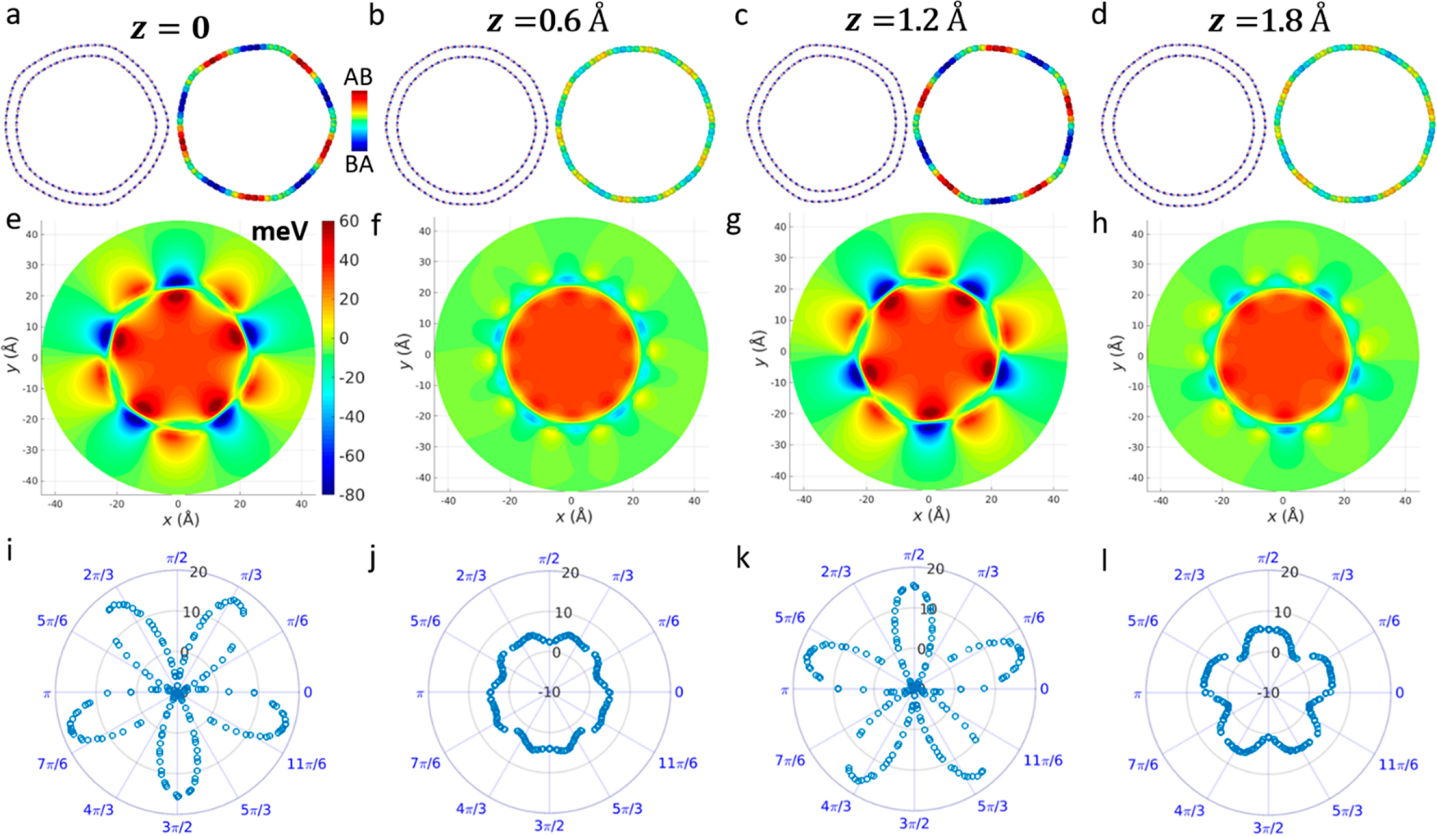
Axial interwall shift induced superstructure and radial polarization variations calculated for the AC (31,31)@(36,36) DWBNNT of preoptimized inner and outer wall diameters of 42.8 and 49.7 Å, respectively. (a–d) Cross-sectional views of the relaxed DWBNNT for several coaxial shifts, *z* = 0, 0.6, 1.2, and 1.8 Å. In each panel, the left illustration represents the atomic structure and the right illustration superimposes the corresponding LPRI map on the outer wall atomic positions (see color bar in panel a). (e–h) DFT calculated electrostatic potential difference maps (with respect to the same individual walls) plotted along the (001) face of the DWBNNTs presented in panels a–d, respectively. The color bar appearing in panel e is common to panels e–h. (i–l) Polar diagrams presenting the difference in electrostatic potential (in meV) calculated outside the tube at a radius of *r* = 35 Å and inside the tube at a radius of *r* = 5 Å. These values are obtained for each angle by radially averaging over a range of 0.02 Å and axially averaging over the length of the unit cell.

Increasing the AC DWBNNT diameter results in more pronounced faceting and higher polarization values. To demonstrate this, we consider next the parallelly stacked AC (46,46)@(51,51) DWBNNT, with an outer wall diameter of 70 Å and an interwall distance of 3.45 Å. Following geometry relaxation, the faceted structure exhibits radial electrostatic potential energy differences of −26 meV in the BA region and 27 meV in the AB region (see SI section 4.2), thus enhancing the slidetronic effect. Conversely, for narrow AC DWBNNTs that exhibit weak faceting, e.g., the parallelly stacked (20,20)@(25,25) DWBNNT with an outer wall diameter of 28 Å, the overall angular polarization anisotropy is considerably reduced (see SI section 4.2). Antiparallelly stacked AC DWBNNTs demonstrate similar facet variations;^30^ however, due to the lack of AB and BA facet stacking configurations, they exhibit considerably smaller and unidirectional polarization variations (see SI section 4.3).

Similar to the ZZ DWBNNT case, the band gap of the parallelly stacked (31,31)@(36,36) DWBNNT exhibits oscillations of ∼0.2 eV, accompanied by work function variations of ∼0.1 eV, upon coaxial displacement (see SI section 4.4). In comparison, for the nonpolar antiparallelly stacked counterpart, we find lower amplitude bandgap variations and similar work function modulations (see SI section 4.4). This indicates that electric polarization has a measurable effect on the bandgap of the faceted DWBNNT considered but a minor effect on its work function.

All examples presented above involve polarization variations in achiral DWBNNTs. Double-walled nanotubes, however, offer a considerably wider variety of structures, differing by the nature of the two walls. In practice, a huge number of structures can be envisioned, as long as two constraints are fulfilled: (i) circumferential frustration is obeyed by appropriate choice of chiral indices, and (ii) the interwall distance should not significantly deviate from the equilibrium interlayer distance of the corresponding 2D bilayer system. If, for example, the two nanotube walls are chiral but share the same chiral angle, a monochiral double-walled nanotube is formed.^29^ An example would be the (120,100)@(126,105) DWBNNT that presents achiral polar domains that vary under axial motion similar to their achiral DWBNNT counterparts (see SI section 5). If, however, the two walls differ in chiral angle, chiral faceted superstructures appear that exhibit screw-like motion upon coaxial interwall sliding.^30^ These will induce chiral polarization pattern variations. To demonstrate this, we consider the parallelly stacked bichiral (70,70)@(77,74) DWBNNT with an outer wall diameter of 104 Å, a chiral angle of 0.657°, and an interwall distance of 3.8 Å (see Figure 3a,b). LPRI analysis reveals clear helical polarization patterns that spiral around the circumference of the DWBNNT (Figure 3c). Upon coaxial interwall sliding the facets exhibit coupled translational and rotational variations that are clearly manifested in the LPRI maps (see SI Movie S3). Similar to the equilateral triangle moiré domains appearing in marginally twisted 2D *h*-BN interfaces,^4^ the polar domains in the bichiral DWBNNT form adjacent extended obtuse isosceles triangles that are expected to exhibit a similar potential drop. Notably, the coaxial sliding potential energy profile appearing in SI section 2 (Figure S2d) predicts negligible sliding potential energy barriers for the bichiral nanotube that are 6–7 orders of magnitude smaller than those exhibited by their achiral AC and ZZ counterparts (Figure S2b,c). This effect, attributed to reduced interwall lattice commensurability, is expected to result in negligible interwall sliding friction,^30^ thus marking bichiral double-walled nanotubes as promising candidates for nano slidetronic devices, such as high-frequency nano generators, switches, and memory components.

**Figure 3:**
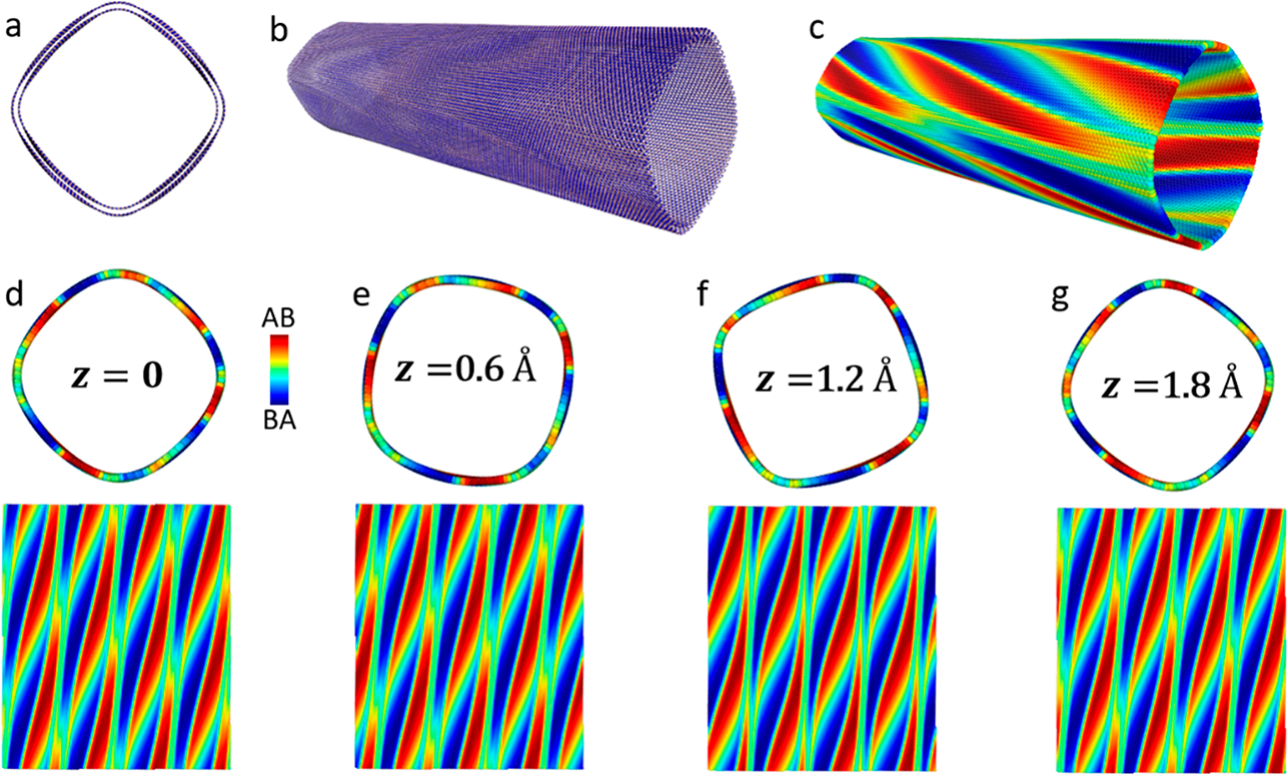
Axial interwall shift induced superstructure and radial polarization variations calculated for the bichiral (70,70)@(77,74) DWBNNT of preoptimized inner and outer wall diameters of 96.7 and 104.3 Å, respectively. (a, b) Cross-sectional and perspective views of the relaxed DWBNNT. (c) LPRI map superimposed on the outer DWBNNT wall (presented in the same perspective as in panel b), exhibiting chiral polarization patterns. (d–g) Cross-sectional (upper subpanels) and unrolled (lower subpanels, see SI section 6) views of the LPRI maps superimposed on the outer wall as obtained for several coaxial shifts, *z* = 0, 0.6, 1.2, and 1.8 (see color bar in panel d). The horizontal (328 Å in length) and vertical (378 Å in length) axes in the 2D LPRI maps correspond to the circumferential and axial directions of the DWBNNTs, respectively, as explained in SI section 6.

The rich polar domain variation physics exhibited by DWBNNTs under coaxial interwall sliding predicted herein constitutes the first demonstration of 1D slidetronics. By controlling the chiral indices of the two tube walls one may design a plethora of DWBNNT structures with predetermined circumferentially faceted super structures.^29,30^ These, in turn, lead to diverse slidetronic characteristics, ranging from strong local variations of the electrostatic potential energy to delocalized chiral polar domain dynamics. Measurement of these predicted effects requires interwall manipulation^27,36−39^ of faceted multiwalled nanotubes^24−29^ and local probing of the resulting polarization variations.^4−6^ Similar to the case of 2D ferroelectric layered materials,^4^ such local probing could also be used to trigger domain wall shifting and induce reversible polarization switching, which could be utilized in memory devices. Furthermore, the intrinsically low interwall friction characteristics of multiwalled nanotubes supports the fabrication of coaxial sliding GHz oscillators.^40−43^ By connecting local probes (e.g., conducting tips) to the outer wall of the oscillator, the periodic local polarization variations could generate AC currents, thus supporting the realization of nanogenerators.

## Methods

All geometry optimizations have been performed using the classical Tersoff^44^ intralayer potential and the dedicated interlayer potential,^33,34^ as implemented in the LAMMPS^45^ package (see further details in SI section 2). Single-point DFT calculations to obtain the electrostatic potential maps have been performed on the relaxed structures using the Vienna Ab initio Simulation Package.^46^ Periodic boundary conditions were applied along the axial direction using a vacuum size of 40 Å along the perpendicular directions to avoid spurious interactions between adjacent nanotube images. The Perdew–Burke–Ernzerhof generalized gradient exchange-correlation density functional approximation^47^ was used along with the scalar-relativistic projector augmented wave description of the core electrons. A plane-wave cutoff energy of 800 eV was used with a *k*-mesh of 1 × 1 × 10 points for armchair DWBNNTs and 1 × 1 × 6 points for zigzag DWBNNTs, using the gamma-centered scheme. Additional polarization mapping was performed on the relaxed structures using the polarization registry index method and its local version that were proposed and explained in detail in an earlier study^35^ and generalized herein to describe curved structures (see further details in SI sections 1 and 6). The comparison between the DFT potential maps and the LPRI maps allowed us to establish a relation between the local interwall lattice registry and local polarization.

## Supporting Information Available

Further details regarding the PRI evaluation; quasi-static structural simulations; AC@AC, ZZ@ZZ, and monochiral DWBNNT polarization calculations; and the 2D LPRI mapping procedure (PDF)
Movie S1: Movie of the ZZ@ZZ DWNTs false colored according to the LPRI map (MP4)
Movie S2: Movie of the AC@AC DWNTs false colored according to the LPRI map (MP4)
Movie S3: Movie of the bichiral DWNTs false colored according to the LPRI map (MP4)
Transparent Peer Review report available (PDF)
