# Supplementary material for: Nanotube Slidetronics
Source: J Phys Chem Lett. 2023 Dec 21;15(1):9–14. doi: 10.1021/acs.jpclett.3c02681 (PMC10788953; doi:10.1021/acs.jpclett.3c02681)
Supplement: Supplementary file 1 — jz3c02681_si_001.pdf [file jz3c02681_si_001.pdf]

Supporting Information

Nanotube Slidetronics

Wei Cao, Michael Urbakh, and Oded Hod*

Department of Physical Chemistry, School of Chemistry, The Raymond and Beverly Sackler Faculty of Exact Sciences and The Sackler Center for Computational Molecular and Materials Science, Tel Aviv University, Tel Aviv 6997801, Israel

*E-mail: urbakh@tauex.tau.ac.il

In this supplementary information, we provide additional details on certain aspects of the study reported in the main text. The following issues are discussed:

S1. Polarization registry index (PRI) 3

S2. Quasi-static simulations 5

S3. Additional details regarding the calculations of ZZ@ZZ DWBNNTs 7

S3.1. Charge redistribution map of parallelly-stacked (55,0)@(63,0) DWBNNT 7

S3.2. Electrostatic polarization of parallelly-stacked ZZ (30,0)@(38,0) DWBNNT 8

S3.3. Electrostatic polarization of anti-parallelly stacked ZZ (55,0)@(63,0) DWBNNT 9

S3.4. Band structure and work function of the parallelly stacked ZZ (55,0)@(63,0) DWBNNT 10

S4. Additional details regarding the calculations of AC@AC DWBNNTs 11

S4.1. Charge redistribution map of parallelly-stacked (31,31)@(36,36) DWBNNT 11

S4.2. Electrostatic polarization of parallelly-stacked AC (20,20)@(25,25) and (46,46)@(51,51) DWBNNTs 12

S4.3. Electrostatic polarization of anti-parallelly stacked AC (31,31)@(36,36) DWBNNT 13

S4.4. Band structures and work functions of the AC (31,31)@(36,36) DWBNNT 14

S5. Electrostatic polarization of mono-chiral DWBNNT 16

S6. 2D LPRI mapping 17

Movie captions 18

References 19

S1. Polarization registry index (PRI)

The polarization registry index (PRI) is a geometric measure that correlates interlayer registry in layered material interfaces and their electric polarization characteristics. A detailed description of the PRI, its global (GPRI) and local (LPRI) definitions, and their performance are provided in Ref. 1. Originally developed for planar bilayer systems, such as hexagonal boron-nitride (h-BN), it can be generalized to describe curved structures, e.g. double walled nanotubes (DWNT). The generalization follows the spirit of the PRI for planar systems consisting of non-trivial sublayer structure, such as transition metal dichalcogenides. Here, the projected overlap between two-dimensional Gaussian functions (the standard deviations of which are marked by σ) associated with atoms residing on adjacent layers is scaled by a factor that depends exponentially (with an exponent α) on the vertical distance between the two. Specifically, for the case of h-BN, Eq. (4) (rather than Eq. (1)) of Ref. 1 is used in Eq. (2) of the same reference for the PRI calculation. The vertical distance between the two atoms is defined according to their local normal vectors following the procedure described in Ref. 2. Parameter refitting (see Table S1) following the fitting procedure described in Ref. 1 for the planar bilayer system, yields excellent agreement between the $GPRI^{h-\mathrm{BN}}$ and density functional theory (DFT) calculated polarization profiles (see Fig. S1).

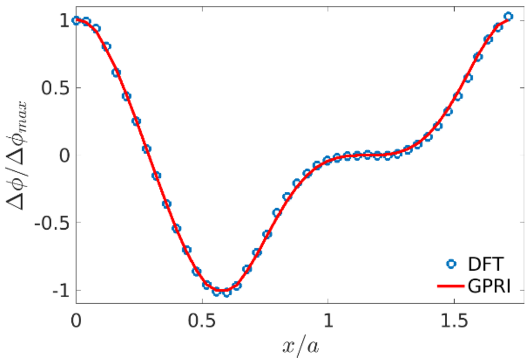

Figure S1. Polarization profiles calculated using DFT (adapted from Ref. 1, open circles) and the generalized $\mathrm{GPRI}^{h-\mathrm{BN}}$ (full lines) along the armchair direction. The x-axis is normalized to the intralayer lattice constant of h-BN $(a = 2.51\text{Å})$.

Table S1. Refitted generalized $GPRI^{h-BN}$ parameters for bilayer h-BN. The effective Gaussian width of each atomic pair appearing in the table (σ_B^N/σ_N^B) is given in units of $a/\sqrt{3}$, where a is the lattice constant ($a_{h-BN} = 2.51$ Å). Average ($\bar{x}$) difference between the normalized reference DFT potential drop profile and the $GPRI^{h-BN}$ results, and the corresponding standard deviation (σ) obtained using the optimal parameter sets are also provided.

***h*-BN**	**Parameter**
$\sigma_B^N(\sigma_N^B)$	0.5
$\alpha(\text{Å}^{-1})$	2.5
$\bar{x}$	0.0061
σ	0.0057

S2. Quasi-static simulations

Quasi-static axial inter-wall sliding simulations were performed in steps of $\Delta z = 0.1$ Å using classical force-fields as described in the methods section. For each inter-wall shift the atomic coordinates of the double-walled boron-nitride nanotube (DWBNNT) structure were first optimized (keeping their axial, z, coordinate fixed) using the Fire algorithm[3] with fixed supercell size. This was followed by an optimization step of the nanotube supercell length using the conjugate gradient (CG) algorithm.[4] This two-step energy minimization procedure was repeated five times, which is sufficient to obtain well converged results (see Fig. S2a). In each repetition, both minimization stages were terminated when the forces acting on each degree of freedom reduced below 10^{-6} eV/Å. Finally, the atomic coordinates were further relaxed using the Fire algorithm with a force tolerance of 10^{-6} eV/Å. A typical diagram presenting the total energy minimization during the relaxation procedure is shown in Fig. S2a for the (70,70)@(77,74) DWBNNT.

The resulting quasi-static sliding energy profiles obtained using our interlayer potential (ILP, see Methods section) are presented in Fig. S2b-d for the (31,31)@(36,36), (55,0)@(63,0), and (70,70)@(77,74) DWBNNTs. We find that the zigzag (ZZ) (55,0)@(63,0) DWBNNT considered presents sliding energy corrugation higher by nearly an order of magnitude than its armchair (AC) (31,31)@(36,36) counterpart, and by six orders of magnitude than the bichiral (70,70)@(77,74) DWBNNT.

The corresponding energy profiles, calculated using single-point DFT calculations (see Methods section) on the ILP relaxed structures of the ZZ (55,0)@(63,0) and (31,31)@(36,36)AC DWBNNTs, are depicted in Figs. S2b-c. The sliding energy corrugations of ~1.1 and ~0.4 meV/atom, respectively, are lower than those obtained using the ILP (3.4 and 0.9 meV/atom, respectively). Nonetheless, considering the overall very small energy landscape corrugation, these differences can be considered to be minor. We note that these sliding energy barriers compare well to those obtained for 2D bilayers of *h*-BN (2.3 meV/atom)[5] and MoS_2 (0.2 meV/atom)[6].

The ILP energy barriers per unit-cell for DWBNNT coaxial sliding are 0.5 eV, 0.1 eV, and 0.2 meV for ZZ, AC, and bichiral DWBNNTs, respectively, calculated based on the above reported values and on the number of atoms per unit cell. We therefore conclude that room temperature fluctuations are insufficient to induce coaxial sliding of the achiral DWBNNTs.

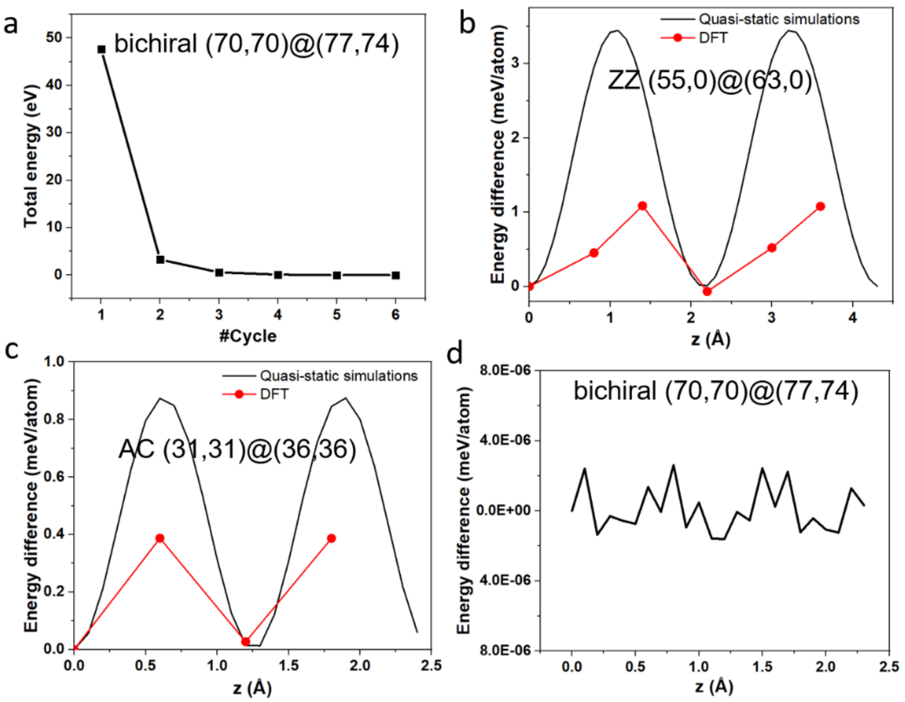

Fig. S2. Quasi-static coaxial sliding energy simulation results. (a) Energy minimization curve for the bichiral (70,70)@(77,74) DWBNNT. (b)-(d) Sliding energy profiles of the ZZ (55,0)@(63,0), AC (31,31)@(36,36), and bichiral (70,70)@(77,74) DWBNNTs, respectively. Single point total energy DFT results (see Methods section) are presented for comparison by the red lines in panels (b), and (c).

S3. Additional details regarding the calculations of ZZ@ZZ DWBNNTs

S3.1. Charge redistribution map of parallelly-stacked (55,0)@(63,0) DWBNNT

In the discussion of Fig. 1 provided in the main text, we attributed the emerging electrostatic potential energy patterns to charge redistribution between the two ZZ (55,0)@(63,0) tube walls. To demonstrate this, we present in Fig. S3 charge density difference maps (with respect to the individual tubes) obtained at different interlayer displacements, corresponding to the structures discussed in the main text. As discussed in the main text, the facetted regions exhibit either AB or BA stacking in this case. This is manifested in the charge redistribution maps as inter-wall charge density difference polarity reversal during the sliding cycle.

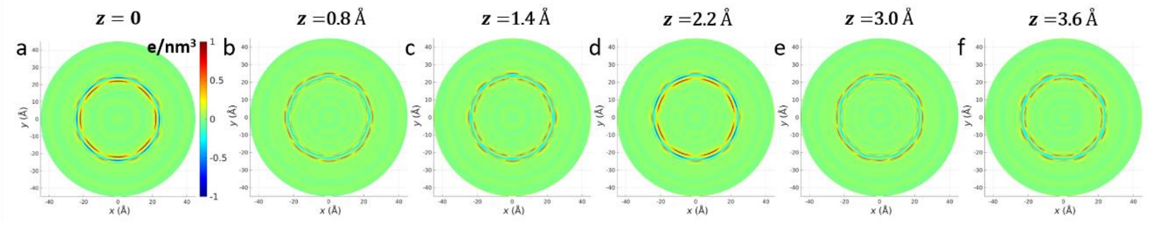

Figure S3. Charge density difference maps (with respect to the same individual walls) plotted along the (001) face of the parallelly-stacked ZZ (55,0)@(63,0) DWBNNT for several coaxial shifts, $z = 0, 0.8, 1.4, 2.2, 3.0,$ and 3.6 Å.

S3.2. Electrostatic polarization of parallelly-stacked ZZ (30,0)@(38,0) DWBNNT

In Fig. 1 of the main text, we present electrostatic polarization results for the ZZ (55,0)@(63,0) DWBNNT. To demonstrate the sensitivity of the results towards the DWBNNT diameter, we consider here the ZZ (30,0)@(38,0) DWBNNT of smaller diameter. Comparing the polarization maps and polar diagrams of the two DWBNNTs prior to coaxial displacement (see Fig. S4 and Fig. 1a), both exhibiting eight-fold symmetry after geometry relaxation, we find that the narrower DWBNNT does not exhibit faceting, leading to lower interwall lattice commensurability, accompanied by overall lower polarization and considerably reduced azimuthal electrostatic potential anisotropy.

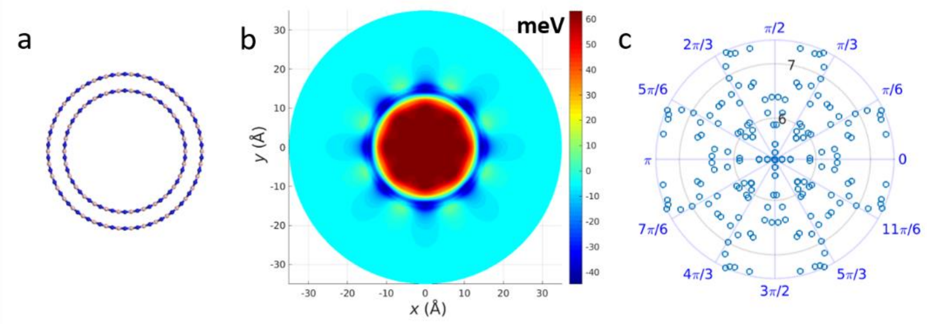

Figure S4. (a) Cross-sectional view of the ZZ (30,0)@(38,0) DWBNNT. (b) DFT calculated electrostatic potential difference maps (with respect to the same individual walls) plotted along the (001) face of the DWBNNT presented in panel a. (c) Polar diagrams presenting the radial electrostatic potential energy drop (in meV) across the DWBNNT presented in panel a.

S3.3. Electrostatic polarization of anti-parallelly stacked ZZ (55,0)@(63,0) DWBNNT

In contrast to the AB/BA stacking domains observed in the facet regions of parallelly-stacked AC DWBNNTs, anti-parallelly stacked AC DWBNNTs (see Fig. S5) exhibit AA' stacked facet regions. Planar AA' stacked *h*-BN bilayers do not exhibit out-of-plane polarization. Correspondingly, the AA' anti-parallelly stacked DWBNNT facet regions exhibit considerably lower polarization values as compared to their AB/BA parallelly-stacked counterparts.

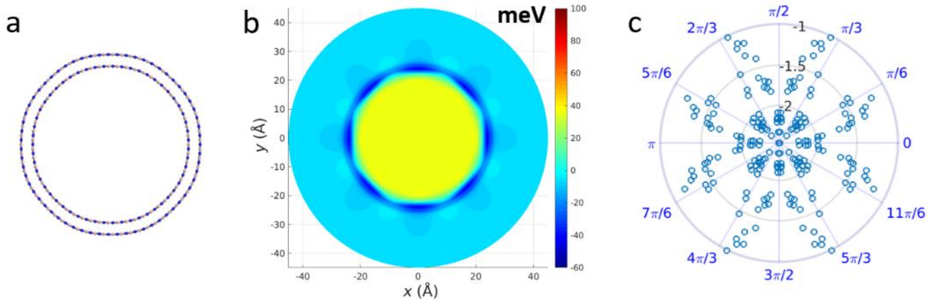

Figure S5. (a) Cross-sectional view of anti-parallelly stacked (55,0)@(63,0) DWBNNT. (b) DFT calculated electrostatic potential difference maps (with respect to the same individual walls) plotted along the (001) face of the DWBNNT presented in panel a. (c) Polar diagrams presenting the radial electrostatic potential energy drop (in meV) across the DWBNNT presented in panel a.

S3.4. Band structure and work function of the parallelly stacked ZZ (55,0)@(63,0) DWBNNT

As mentioned in the main text, Figs. S6a, b display respectively the DFT (see Methods section) bandgap and band diagram of parallelly-stacked optimized ZZ (55,0)@(63,0) DWBNNT as a function of coaxial shift. The bandgap exhibits periodic oscillations of ~0.4 eV, and similar to previous reports for smaller diameter DWBNNTs[7] is of direct nature regardless of interwall displacement. Fig. S6c displays work function variations of ~0.1 eV as a function of interwall coaxial displacement.

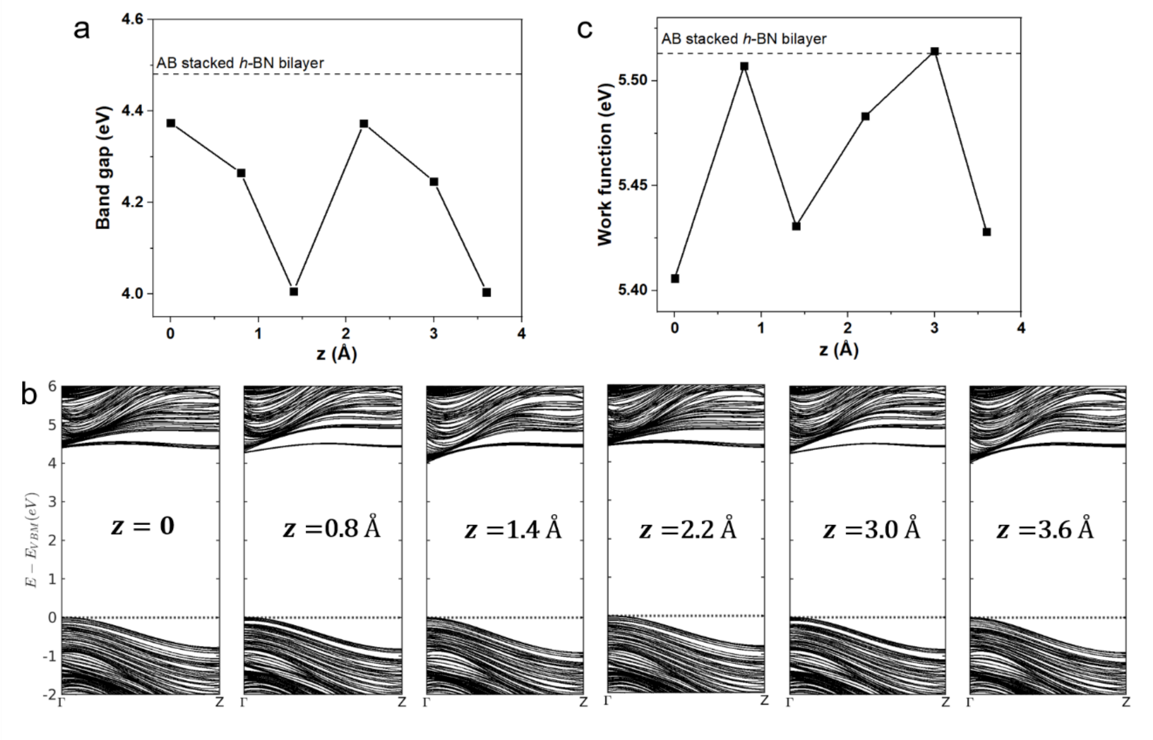

Figure S6. (a) DFT calculated (see Methods section) bandgap of parallelly-stacked ZZ (55,0)@(63,0) DWBNNTs as a function of coaxial interwall displacement. The horizontal dashed line represents the band gap of AB stacked *h*-BN bilayer calculated at the same level of theory. (b) The corresponding band structure obtained at various coaxial interwall displacements. The top of the valence band is set to zero in all panels. (c) Work function variations as a function of coaxial interwall displacement. The horizontal dashed line represents the work function of AB stacked *h*-BN bilayer calculated with the same level of theory.

S4. Additional details regarding the calculations of AC@AC DWBNNTs

S4.1. Charge redistribution map of parallelly-stacked (31,31)@(36,36) DWBNNT

In the discussion of Fig. 2 provided in the main text, we attributed the emerging electrostatic potential energy patterns to charge redistribution between the two AC (31,31)@(36,36) tube walls. To demonstrate this, we present in Fig. S7 charge density difference maps (with respect to the individual tubes) obtained at different interlayer displacements, corresponding to the structures discussed in the main text. We find that for the facetted structures, the charge density redistribution becomes more pronounced (see Fig. S7a and c). This can be attributed to the increased inter-wall lattice commensurability at the facet regions. Notably, the AB and BA stacking regions in each facet show opposite charge difference polarity, and locally asymmetric patterns due to circumferential frustration. These results are in line with the polarization maps presented in the main text.

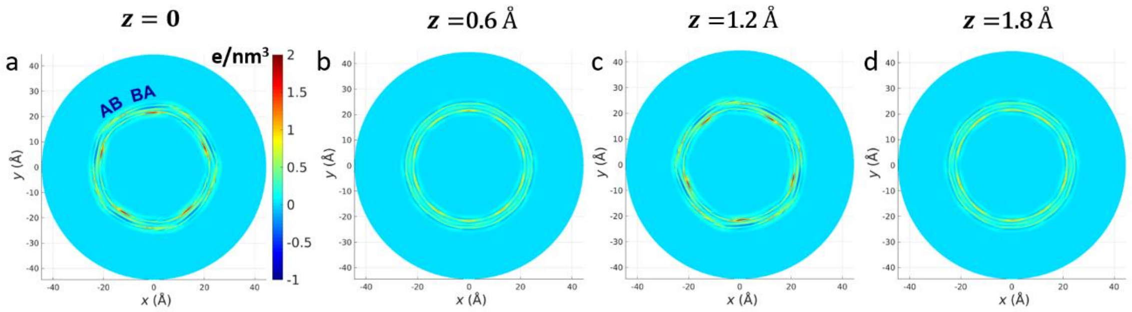

Figure S7. Charge density difference maps (with respect to the same individual walls) plotted along the (001) face of the parallelly-stacked AC (31,31)@(36,36) DWBNNT for several coaxial shifts, $z = 0, 0.6, 1.2,$ and 1.8 Å.

S4.2. Electrostatic polarization of parallelly-stacked AC (20,20)@(25,25) and (46,46)@(51,51) DWBNNTs

In Fig. 2 of the main text, we present electrostatic polarization results for the AC (31,31)@(36,36) DWBNNT. To demonstrate the sensitivity of the results towards the DWBNNT diameter, we consider here two other AC DWBNNTs, including the larger diameter (46,46)@(51,51) and the smaller diameter (20,20)@(25,25) DWBNNTs. Comparing the polarization maps and polar diagrams of the three DWBNNTs prior to coaxial displacement (see Fig. S8 and Fig. 2), all exhibiting five-fold faceting symmetry after geometry relaxation, we find that increasing the tube diameter, results in more pronounced and extended facetted regions of increased inter-wall lattice commensurability, accompanied by enhanced azimuthal electrostatic potential anisotropy.

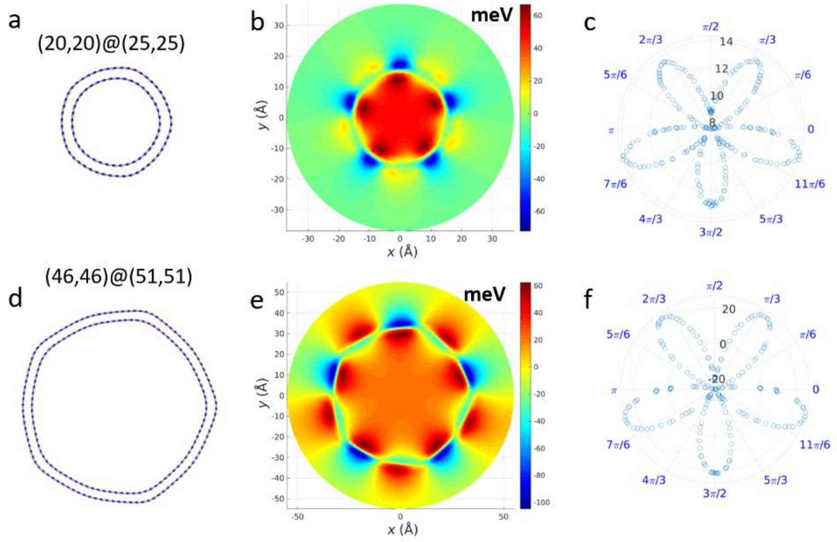

Figure S8. (a) Cross-sectional view of the AC (20,20)@(25,25) DWBNNT. (b) DFT calculated electrostatic potential difference maps (with respect to the same individual walls) plotted along the (001) faces of the DWBNNT presented in panel a. (c) Polar diagrams presenting the radial electrostatic potential energy drop (in meV) across the DWBNNT presented in panel a. (d)-(f) same as a-c for the AC (46,46)@(51,51).

S4.3. Electrostatic polarization of anti-parallelly stacked AC (31,31)@(36,36) DWBNNT

In contrast to the AB/BA stacking domains observed in the facetted regions of parallelly-stacked AC DWBNNTs, anti-parallelly stacked AC DWBNNTs (see Fig. S9) exhibit AA' stacked facet regions. Planar AA' stacked *h*-BN bilayers do not exhibit out-of-plane polarization. Correspondingly, the AA' anti-parallelly stacked DWBNNT facet regions exhibit considerably lower polarization values as compared to their AB/BA parallelly-stacked counterparts.

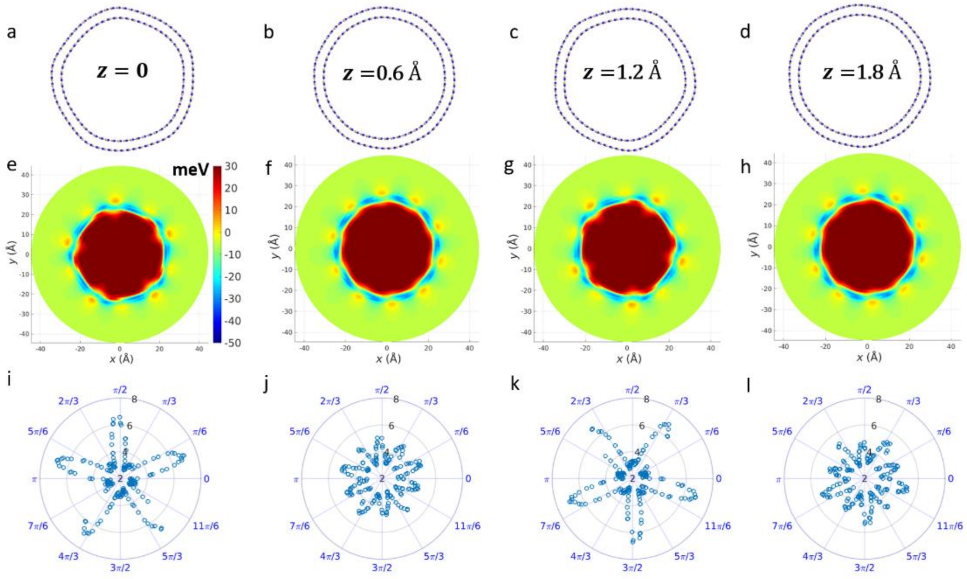

Figure S9. Axial interwall shift induced superstructure and radial polarization variations calculated for the anti-parallelly stacked AC (31,31)@(36,36) DWBNNT. (a)-(d) Cross-sectional views of the relaxed DWBNNT for several coaxial shifts, $z = 0, 0.6, 1.2,$ and 1.8 Å. (e)-(h) DFT calculated electrostatic potential difference maps (with respect to the same individual walls) plotted along the (001) face of the DWBNNTs presented in panels a-d, respectively. The color bar appearing in panel e is common to panels f-h. (i)-(l) Polar diagrams presenting the radial electrostatic potential energy drop (in meV) across the DWBNNTs presented in panels a-d, respectively.

S4.4. Band structures and work functions of the AC (31,31)@(36,36) DWBNNT

As mentioned in the main text, Fig. S10a, b and c display the DFT (see Methods section) band gaps of both parallelly- and anti-parallelly-stacked optimized AC (31,31)@(36,36) DWBNNTs as a function of coaxial displacement, and the corresponding band structure variations. Similar to previous reports for smaller diameter DWBNNTs,[7] AC tubes exhibit indirect band gaps, regardless of interwall shift. Compared to the polar parallelly stacked DWBNNT, the band gap of the non-polar anti-parallelly stacked DWBNNT exhibits similar oscillations but with a reduced amplitude, indicative of the role played by electric polarization on the bandgap of the system. Fig. S10d displays the work function as a function of coaxial interwall displacement, showing similar oscillation amplitude and period for the parallelly and anti-parallelly stacked DWBNNTs. This indicates that local polarization has a minor effect on the work function of the studied DWBNNT.

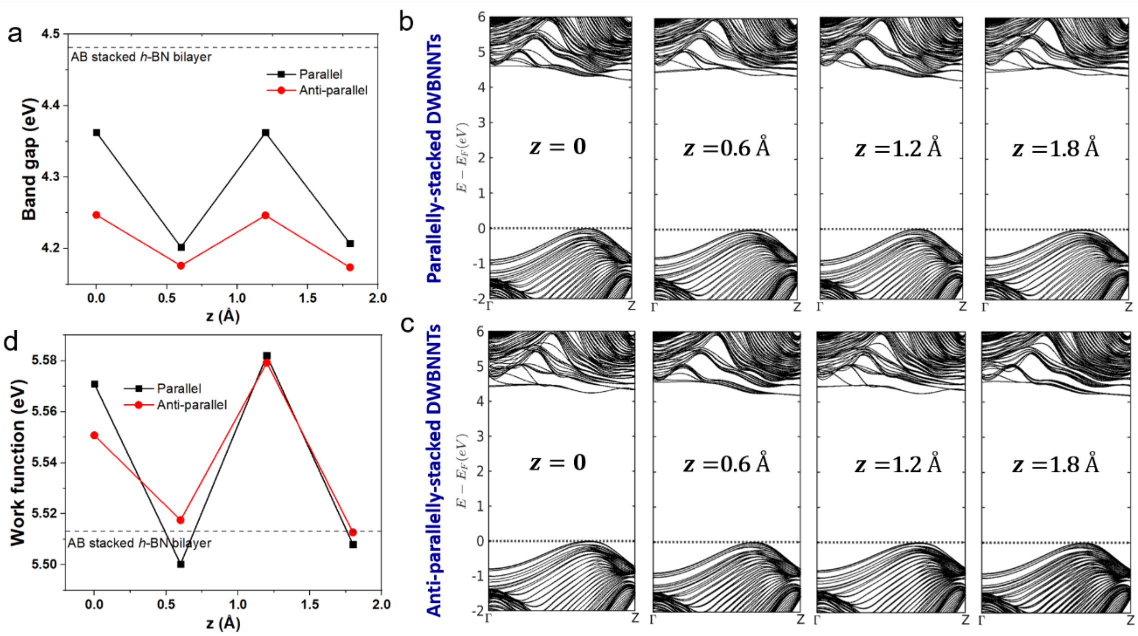

Figure S10. (a) DFT calculated (see Methods section) bandgaps of parallelly-stacked (black) and anti-parallelly-stacked (red) AC (31,31)@(36,36) DWBNNTs. The horizontal dashed line represents the bandgap of AB stacked *h*-BN bilayer calculated at the same level of theory. (b) and (c) show the band structures of parallelly-stacked and anti-parallelly-stacked DWBNNTs at various interwall coaxial displacements, respectively. The top of the valence band is set to zero in all panels. (d) Work function of parallelly-stacked (black) and anti-parallelly-stacked (red) AC (31,31)@(36,36) DWBNNTs at various coaxial interwall displacements. The horizontal dashed line represents the work function of AB stacked *h*-BN bilayer calculated at the same level of theory.

S5. Electrostatic polarization of mono-chiral DWBNNT

To demonstrate electric polarization patterns in parallelly-stacked mono-chiral DWBNNTs we considered the (120,100)@(126,105) system with an unrelaxed outer wall diameter of 160 Å, and an interwall distance of 3.8 Å (see Fig. S11a, b). The LPRI analysis reveals striped polarization patterns distributed along the DWBNNT circumference (see Fig. S11c). Upon coaxial inter-wall sliding, the facets display rotational variations that are clearly visible in the LPRI maps (see Fig. S11d-g). In contrast to bichiral DWBNNTs (see Fig. 3 of the main text), monochiral structures do not exhibit facets and polarization patterns of chiral nature.

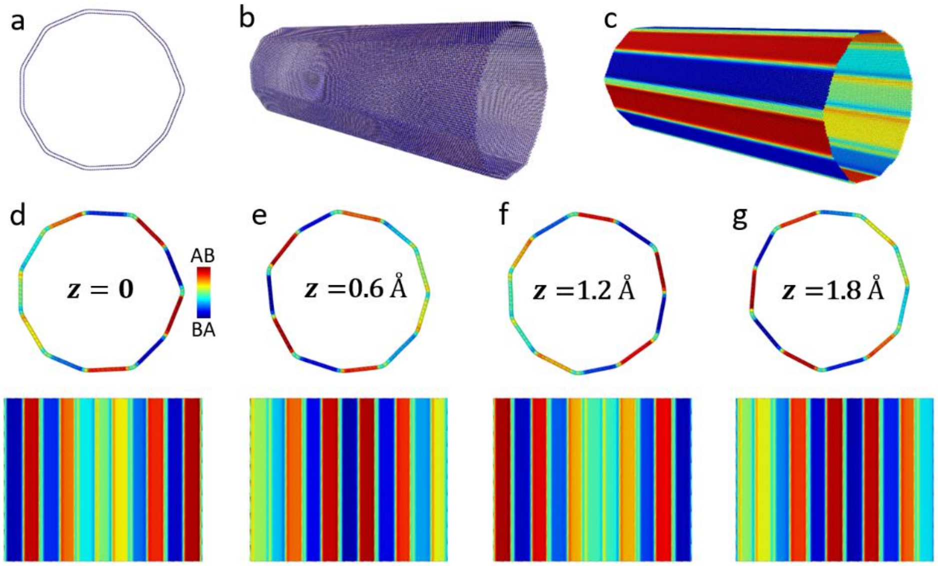

Figure S11. Axial interwall shift induced superstructure and radial polarization variations calculated for the monochiral (120,100)@(126,105) DWBNNT of preoptimized inner and outer wall diameters 152.1 Å and 159.7 Å, respectively. (a), (b) Cross-sectional and perspective views of the relaxed DWBNNT. (c) LPRI map superimposed on the outer wall of panel b, exhibiting achiral polarization patterns. (d)-(g) Cross-sectional (upper sub-panels) and unrolled (lower sub-panels) views of the LPRI maps superimposed on the outer wall as obtained for several coaxial shifts, $z = 0, 0.6, 1.2,$ and 1.8 Å (see color bar in panel d). The horizontal (502 Å in length) and vertical (414 Å in length) axes in the 2D LPRI maps correspond to the circumferential and axial directions of the DWBNNTs, respectively, as explained in SI section 6.

S6. 2D LPRI mapping

The 2D LPRI mapping procedure involves three steps: (i) The LPRI map of the outer wall of the facetted DWBNNT is computed using the method outlined in SI section 1;[1] (ii) The outer wall of the initial unrelaxed DWBNNT is unrolled to a planar surface;[8] (iii) each atom in the unrolled outer wall is assigned its calculated LPRI value from (i).

Movie captions

Movie S1: Movie of the ZZ@ZZ DWNTs false colored according to the LPRI map during coaxial interwall sliding

Movie S2: Movie of the AC@AC DWNTs false colored according to the LPRI map during coaxial interwall sliding

Movie S3: Movie of the bichiral DWNTs false colored according to the LPRI map during coaxial interwall sliding

References

(1). Cao, W.; Hod, O.; Urbakh, M. Interlayer Registry Dictates Interfacial 2D Material Ferroelectricity. *ACS Appl. Mater. Interfaces* **2022,** *14* (51), 57492-57499.

(2). Leven, I.; Guerra, R.; Vanossi, A.; Tosatti, E.; Hod, O. Multiwalled nanotube faceting unravelled. *Nat. Nanotechnol.* **2016,** *11* (12), 1082-1086.

(3). Guénolé, J.; Nöhring, W. G.; Vaid, A.; Houllé, F.; Xie, Z.; Prakash, A.; Bitzek, E. Assessment and optimization of the fast inertial relaxation engine (fire) for energy minimization in atomistic simulations and its implementation in lammps. *Comput. Mater. Sci.* **2020,** *175*, 109584.

(4). Hestenes, M. R.; Stiefel, E. Methods of conjugate gradients for solving linear systems. *J. Res. Natl. Inst. Stand. Technol.* **1952,** *49* (6), 409-436.

(5). Li, L.; Wu, M. Binary compound bilayer and multilayer with vertical polarizations: Two-dimensional ferroelectrics, multiferroics, and nanogenerators. *ACS Nano* **2017,** *11* (6), 6382-6388.

(6). Cao, W.; Hod, O.; Urbakh, M. Interlayer registry index of layered transition metal dichalcogenides. *J. Phys. Chem. Lett.* **2022,** (13), 3353-3359.

(7). Jhi, S.-H.; Roundy, D. J.; Louie, S. G.; Cohen, M. L. Formation and electronic properties of double-walled boron nitride nanotubes. *Solid State Commun.* **2005,** *134* (6), 397-402.

(8). Hod, O. Quantifying the stacking registry matching in layered materials. *Isr. J. Chem.* **2010,** *50* (4), 506-514.
