# Supplementary material for: Nanotube Slidetronics
Source: J Phys Chem Lett. 2023 Dec 21;15(1):9–14. doi: 10.1021/acs.jpclett.3c02681 (PMC10788953; doi:10.1021/acs.jpclett.3c02681)
Supplement: Supplementary file 5 — jz3c02681_si_005.pdf [file jz3c02681_si_005.pdf]

jz-2023-02681c.R1

Name: Peer Review Information for "Nanotube Slidetronics"

First Round of Reviewer Comments

Reviewer: 1

Comments to the Author

The authors have conducted a systematic investigation into one-dimensional slidetronics within double-walled boron-nitride nanotubes, where local electrostatic polarization patterns are influenced by the nanotube walls and their relative arrangement. The manuscript is intriguing and warrants further enhancement before publication.

1) Have the authors taken into account the role of van der Waals interactions in their calculations? These interactions could significantly impact the bonding between the two nanotubes.

2) Could the authors provide a more detailed explanation of their methodology for calculating polarization?

3) From an experimental perspective, what are the practical considerations and challenges in synthesizing these sliding double-walled boron-nitride nanotubes (DWBNBTs), and how can the control of their relative angles be achieved?

4) Have the authors explored the use of more precise machine learning interatomic potentials in their research?

The authors could consider investigating the influence of different factors, such as temperature and external electric fields, on the slidetronic behavior of the nanotubes.

Reviewer: 2

Comments to the Author

The manuscript entitled " Nanotube Slidetronics" reported that double-walled boron-nitride nanotubes as One-dimensional slidetronics. However, some critical questions need to be clarified before I can recommend the publication of the present manuscript in The Journal of Physical Chemistry Letters, which undergo major revision and meet the journal criteria by considering the following comments and suggestions.

1. The title of this manuscript should change to be more specific and precise, e.g. including information of “Double-walled Boron-nitride Nanotube Slidetronics”.

2. The abstract needs to be rewritten to give the main conclusions of this article.

3. Several misspellings are found in the references part (i.e., name of journals as well as of authors).

This work represents the theoretical demonstration of slip characteristics in one-dimensional double-walled boron nitride carbon nanotubes, revealing complex physical properties. The researchers found that the local electrostatic polarization pattern along the nanotube body is influenced by the properties of both nanotube walls, their relative configuration, and the azimuthal surface modulation during coaxial inner-wall sliding. Furthermore, the article briefly discusses the potential applications of this sliding electron effect, especially in low-dimensional nanogenerators.

Here are a few more questions ：

1. What is the role of the chirality index in this study, and how can an appropriate chirality index be chosen to generate a helical chiral polarization pattern?

2. For the study of double-walled boron nitride carbon nanotubes, what experimental methods and techniques are used to validate and further explore this phenomenon?

Author's Response to Peer Review Comments:

Dear Prof. Editor,

We thank you for communicating with us the review reports on our manuscript (jz-2023-02681c) titled “Nanotube slidetronics”, recently submitted for your consideration for publication in *The Journal of Physical Chemistry Letters*. We also want to express our sincere appreciation to the referees for providing valuable comments and suggestions that have helped us in improving our manuscript.

Attached to this resubmission, please find our point-by-point response to all the comments raised by the referees and their corresponding suggestions. Also, please find attached the revised manuscript, including the highlighted modifications.

We believe that our responses have adequately addressed the referees’ comments and we hope that you will share our view that our manuscript, in its revised form, merits publication in *The Journal of Physical Chemistry Letters*.

Sincerely yours,

Prof. Michael Urbakh

School of Chemistry,

Tel Aviv University

Tel Aviv 6997801, Israel

Reviewer: 1

"The authors have conducted a systematic investigation into one-dimensional slidetronics within double-walled boron-nitride nanotubes, where local electrostatic polarization patterns are influenced by the nanotube walls and their relative arrangement. The manuscript is intriguing and warrants further enhancement before publication."

Response:

We thank the reviewer for the positive evaluation of our manuscript.

1) Have the authors taken into account the role of van der Waals interactions in their calculations? These interactions could significantly impact the bonding between the two nanotubes.

Response:

We thank the reviewer for raising this important point. The geometry optimization to obtain the faceted nanotube structures has been performed using the anisotropic interlayer potential (ILP) developed in our group. This potential accounts explicitly for van der Waals interactions and was parametrized against state-of-the-art non-local many-body dispersion (MBD-NL) corrected DFT calculations.[1-2] Indeed, should we have not appropriately accounted for long-range dispersive interactions, the facetted structure would not have been obtained.

2) Could the authors provide a more detailed explanation of their methodology for calculating polarization?

Response:

To evaluate the polarization of the facetted nanotubes, we have performed single-point DFT calculations on the ILP relaxed structures using the Vienna Ab initio Simulation Package. The electrostatic potential maps were obtained using periodic boundary condition, applied along the axial direction using a vacuum size of 40 Å along the perpendicular directions to avoid spurious interactions between adjacent nanotube images. The Perdew−Burke−Ernzerhof generalized gradient exchange−correlation density functional approximation was used along with the scalar-relativistic projector augmented wave description of the core electrons. A plane-wave cutoff energy of 800 eV was used with a k-mesh of 1 × 1 × 10 points for armchair DWBNNTs and 1 × 1 × 6 points for zigzag DWBNNTs, using the gamma-centered scheme.

In addition to the first-principles calculations, we have also performed polarization mapping using the polarization registry index (PRI) methods that was proposed and explained in details in an earlier study.[3] As described in the Ref. 3, the PRI is a physically intuitive and highly computationally efficient geometric approach to characterize the interfacial polarization in a variety of homogeneous and heterogeneous

layered interfaces, including *h*-BN, as well as hexagonal and non-hexagonal TMDs. In the present study, the PRI approach, and its local version (termed the LPRI), were generalized to describe curved structures, as discussed in SI section S1. Practically, the LPRI is a local commensurability atomic measure that scales from -1 to 1, representing BA to AB stacking modes.

The comparison between the DFT potential maps and the LPRI maps allowed us to establish a relation between local interwall lattice registry and local polarization.

We note that in the case of chiral nanotubes, where the large unit cell prohibits explicit first-principles polarization calculations, the simplistic LPRI approach allowed us to characterize the local polarization patterns of the system.

The Methods section in the revised manuscript was modified to better reflect these points.

3) From an experimental perspective, what are the practical considerations and challenges in synthesizing these sliding double-walled boron-nitride nanotubes (DWBNBTs), and how can the control of their relative angles be achieved?

Response:

Interwall faceting has been experimentally observed in many nanotube architectures (see, e.g., Refs. 4-9). Furthermore, gaining control over BNNT shell helicity can be achieved through advanced production methods, as summarized in the review of Ref. 10. In addition, interwall sliding in DWBNNTs has been achieved experimentally both for coaxial pull-out and retraction displacements[11-13] and for interwall twisting.[7, 14] Hence, all ingredients required to observe the proposed phenomenon are already experimentally available.

This is emphasized in the concluding paragraph of the main text stating that: "Measurement of these predicted effects requires the experimental interwall manipulation[27, 35-38] of facetted multi-walled nanotubes[24-29] and local probing of the resulting polarization variations.[4-6] Furthermore, the intrinsically low inter-wall friction characteristics of multi-walled nanotubes supports the fabrication of coaxial sliding GHz oscillators[39-42] that, when coupled to the local polarization variations discovered herein, can serve as high-frequency nano-generators, switches, and memory devices."

4) Have the authors explored the use of more precise machine learning interatomic potentials in their research?

Response:

In the present study, all geometry optimizations to obtain the faceted nanotube structures have been performed using the anisotropic interlayer potential (ILP) developed in our group. This potential was parametrized against state-of-the-art non-local many-body dispersion (MBD-NL) corrected DFT calculations,[1-2] and was found

to reliably and accurately describe the interlayer interactions in layered material interfaces.[9, 15-18]

We are presently developing machine learning potentials, trained against similar reference datasets, for defected graphene structures. As part of this effort, we are benchmarking our machine learning approach against pristine interface systems. Notably, we find no advantage of machine learning potentials over classical ILPs for the case of pristine interfaces. In fact, the former are much more computationally demanding with no significant physical gain. For defected systems the case is different, but this is out of the scope of the present paper and is to be discussed in future publications.

The authors could consider investigating the influence of different factors, such as temperature and external electric fields, on the slidetronic behavior of the nanotubes.

Response:

We thank the referee for this valuable suggestion. Indeed, thermal fluctuations and the application of external fields may influence both the structure of facetted nanotubes and their polarization patterns. In the present study, we opted to focus on the new phenomenon of 1D polarization and slidetronic ferroelectricity and explore its basic characteristics. In order to avoid defocusing of the manuscript, and after deliberating the reviewer's suggestion, we have decided to defer the discussion of thermal and external field effects to future publications.

Reviewer: 1

The manuscript entitled " Nanotube Slidetronics" reported that double-walled boron-nitride nanotubes as One-dimensional slidetronics. However, some critical questions need to be clarified before I can recommend the publication of the present manuscript in The Journal of Physical Chemistry Letters, which undergo major revision and meet the journal criteria by considering the following comments and suggestions.

Response:

We thank the reviewer for carefully reviewing our manuscript.

1. The title of this manuscript should change to be more specific and precise, e.g. including information of "Double-walled Boron-nitride Nanotube Slidetronics".

Response:

We thank the reviewer for the kind suggestion for an alternative title. While the suggested title is more specific, we do prefer to keep the original title, since the discovered phenomenon, demonstrated herein for BNNTs, is of general nature and is expected to be observed in any non-centrosymmetrically stacked facetted multi-walled nanotube surface. Since this is more a matter of taste rather than a scientific remark, we believe that this should not be considered as a major issue.

2. The abstract needs to be rewritten to give the main conclusions of this article.

Response:

This, again, is a stylistic preference. In our view, while being concise, the abstract does provide all required information both on the main findings and on the main conclusions.

3. Several misspellings are found in the references part (i.e., name of journals as well as of authors).

Response:

We thank the referee for pointing out some typos in the references list. We have carefully checked the list and have found no further typos in the revised manuscript.

This work represents the theoretical demonstration of slip characteristics in one-dimensional double-walled boron nitride carbon nanotubes, revealing complex physical properties. The researchers found that the local electrostatic polarization pattern along the nanotube body is influenced by the properties of both nanotube walls, their relative configuration, and the azimuthal surface modulation during coaxial inner-wall sliding. Furthermore, the article briefly discusses the potential applications of this sliding electron effect, especially in low-dimensional nanogenerators.

Here are a few more questions:

1. What is the role of the chirality index in this study, and how can an appropriate chirality index be chosen to generate a helical chiral polarization pattern?

Response:

We thank the referee for raising this interesting point. In the present study, we did not define any chirality index. Maybe the reviewer refers to the chiral indices of the tube walls. The chiral indices dictate the superstructure of the facetted nanotube, as was discussed in details in Refs. 9 and 15. In turn, the local registry at each facet dictates the local polarization profile, as demonstrated in the present study. For example, if one wants to produce achiral axial polarization patterns, one should choose two walls of the same chiral angle. If, on the other hand, one is interested in helical polarization patterns, the chiral angles of the walls should differ, and the helicity can be controlled by the corresponding chiral angle difference.

This is conveyed in the concluding paragraph of the main text, where we state the following: "By controlling the chiral indices of the two tube walls one may design a plethora of DWBNNT structures with pre-determined circumferentially facetted super structures.". To make the point clearer we added references to the two papers mentioned above that demonstrate how one can control the faceting patterns via the chiral angles of adjacent nanotube walls.

2. For the study of double-walled boron nitride carbon nanotubes, what experimental methods and techniques are used to validate and further explore this phenomenon?

Response:

Interwall faceting has been experimentally observed in many nanotube architectures (see, e.g., Refs. 4-9). Furthermore, gaining control over BNNT shell helicity can be achieved through advanced production methods, as summarized in the review of Ref. 10. In addition, interwall sliding in DWBNNTs has been achieved experimentally both for coaxial pull-out and retraction displacements[11-13] and for interwall twisting.[7, 14] Once all these ingredients are put together, local probing techniques, such as those used in Refs. 19-21, can be used to characterize the surface polarization patterns.

This is emphasized in the concluding paragraph of the main text stating that: "Measurement of these predicted effects requires the experimental interwall manipulation[27, 35-38] of facetted multi-walled nanotubes[24-29] and local probing of the resulting polarization variations.[4-6] Furthermore, the intrinsically low inter-wall friction characteristics of multi-walled nanotubes supports the fabrication of coaxial sliding GHz oscillators[39-42] that, when coupled to the local polarization variations discovered herein, can serve as high-frequency nano-generators, switches, and memory devices."

References:

(1). Leven, I.; Azuri, I.; Kronik, L.; Hod, O. Inter-layer potential for hexagonal boron nitride. *J. Chem. Phys.* **2014,** *140* (10), 104106.
(2). Maaravi, T.; Leven, I.; Azuri, I.; Kronik, L.; Hod, O. Interlayer potential for homogeneous graphene and hexagonal boron nitride systems: reparametrization for many-body dispersion effects. *J. Phys. Chem. C* **2017,** *121* (41), 22826-22835.
(3). Cao, W.; Hod, O.; Urbakh, M. Interlayer registry dictates interfacial 2D material ferroelectricity. *ACS Appl. Mater. Interfaces* **2022,** *14* (51), 57492-57499.
(4). Gogotsi, Y.; Libera, J. A.; Kalashnikov, N.; Yoshimura, M. Graphite polyhedral crystals. *Science* **2000,** *290* (5490), 317-320.
(5). Zhang, G.; Jiang, X.; Wang, E. Tubular graphite cones. *Science* **2003,** *300* (5618), 472-474.
(6). Celik-Aktas, A.; Zuo, J.-M.; Stubbins, J. F.; Tang, C.; Bando, Y. Double-helix structure in multiwall boron nitride nanotubes. *Acta Crystallogr. A* **2005,** *61* (6), 533-541.
(7). Garel, J.; Leven, I.; Zhi, C. Y.; Nagapriya, K. S.; Popovitz-Biro, R.; Golberg, D.; Bando, Y.; Hod, O.; Joselevich, E. Ultrahigh Torsional Stiffness and Strength of Boron Nitride Nanotubes. *Nano Lett.* **2012,** *12* (12), 6347-6352.
(8). Schouteden, K.; Volodin, A.; Li, Z.; Van Haesendonck, C. Atomically resolved Moire-type superstructures in double-walled carbon nanotubes. *Carbon* **2013,** *61*, 379-385.
(9). Leven, I.; Guerra, R.; Vanossi, A.; Tosatti, E.; Hod, O. Multiwalled nanotube faceting unravelled. *Nat. Nanotechnol.* **2016,** *11* (12), 1082-1086.
(10). Golberg, D.; Bando, Y.; Tang, C. C.; Zhi, C. Y. Boron nitride nanotubes. *Adv. Mater.* **2007,** *19* (18), 2413-2432.
(11). Yu, M.-F.; Lourie, O.; Dyer, M. J.; Moloni, K.; Kelly, T. F.; Ruoff, R. S. Strength and breaking mechanism of multiwalled carbon nanotubes under tensile load. *Science* **2000,** *287* (5453), 637-640.
(12). Zhang, R.; Ning, Z.; Zhang, Y.; Zheng, Q.; Chen, Q.; Xie, H.; Zhang, Q.; Qian, W.; Wei, F. Superlubricity in centimetres-long double-walled carbon nanotubes under ambient conditions. *Nat. Nanotechnol.* **2013,** *8* (12), 912-916.
(13). Niguès, A.; Siria, A.; Vincent, P.; Poncharal, P.; Bocquet, L. Ultrahigh interlayer friction in multiwalled boron nitride nanotubes. *Nat. Mater.* **2014,** *13* (7), 688-693.
(14). Cohen-Karni, T.; Segev, L.; Srur-Lavi, O.; Cohen, S. R.; Joselevich, E. Torsional electromechanical quantum oscillations in carbon nanotubes. *Nat. Nanotechnol.* **2006,** *1* (1), 36-41.
(15). Guerra, R.; Leven, I.; Vanossi, A.; Hod, O.; Tosatti, E. Smallest archimedean screw: facet dynamics and friction in multiwalled nanotubes. *Nano Lett.* **2017,** *17* (9), 5321-5328.
(16). Song, Y.; Mandelli, D.; Hod, O.; Urbakh, M.; Ma, M.; Zheng, Q. Robust microscale superlubricity in graphite/hexagonal boron nitride layered heterojunctions. *Nat. Mater.* **2018,** *17* (10), 894-899.
(17). Gao, X.; Ouyang, W.; Urbakh, M.; Hod, O. Superlubric polycrystalline graphene interfaces. *Nat. Commun.* **2021,** *12* (1), 5694.
(18). Ouyang, W.; Sofer, R.; Gao, X.; Hermann, J.; Tkatchenko, A.; Kronik, L.; Urbakh, M.; Hod, O. Anisotropic interlayer force field for transition metal dichalcogenides: the case of molybdenum disulfide. *J. Chem. Theory Comput.* **2021,** *17* (11), 7237-7245.
(19). Vizner Stern, M.; Waschitz, Y.; Cao, W.; Nevo, I.; Watanabe, K.; Taniguchi, T.; Sela, E.; Urbakh, M.; Hod, O.; Ben Shalom, M. Interfacial ferroelectricity by van der

Waals sliding. *Science* **2021,** *372* (6549), 1462-1466.
(20). Yasuda, K.; Wang, X.; Watanabe, K.; Taniguchi, T.; Jarillo-Herrero, P. Stacking-engineered ferroelectricity in bilayer boron nitride. *Science* **2021,** *372* (6549), 1458-1462.
(21). Woods, C. R.; Ares, P.; Nevison-Andrews, H.; Holwill, M. J.; Fabregas, R.; Guinea, F.; Geim, A. K.; Novoselov, K. S.; Walet, N. R.; Fumagalli, L. Charge-polarized interfacial superlattices in marginally twisted hexagonal boron nitride. *Nat. Commun.* **2021,** *12* (1), 347.

jz-2023-02681c.R2

Name: Peer Review Information for "Nanotube Slidetronics"

Second Round of Reviewer Comments

Reviewer: 3

Comments to the Author

In this manuscript, the authors predicted one-dimensional slidetronics in double-walled boron-nitride nanotubes, which the change of local electrostatic polarization was studied by controlling the chirality, wall diameter and wall slip distance of DWBNNT. This work was interesting. But I have several comments before recommending this work for publication in the Journal of Physical Chemistry Letters.

1) The abstract is too brief and cannot clearly express the core content of the manuscript, which is not conducive to readers' reading. And the author does not specify how parallel and anti-parallel are defined. Meanwhile, its full name should be written when the abbreviation DWBNNT the first appears. In addition, some references cannot be found, such as Ref. 31.

2) The article was mentioned ferroelectricity at the beginning, but the author only discusses local electrostatic polarization of DWBNNT later. The polarization of double-layer h-BN slip without bended is reversible. Is it possible to be flipped polarize of the DWBNNT?

3) How to realize the slip electronics of DWBNNT experimentally? Is the electrical polarity generated by this slip reliable? What are its potential applications?

Reviewer: 4

Comments to the Author

The work reports variations in hBN stacking across the body/area of two concentric BNNT’s. Stacking variations in turn, as well discussed in the literature for planar hBN bilayers, a trendy topic, cause minor variation of polarization (normal component. This is what the authors explore and map. The group is reputable and known for reliable BN-BN potentials as well as superlubricity for incommensurate situations, both relevant to this study. So overall results are nice, qualitatively intuitive, no surprises, and quantitatively can be trusted. One thing I am not sure what/how are those nanogenerator and memory

devices will work!? What is generated? What/how is switched? How the “bits” are written in and read-put in memory case? Conceptually, in principle, never mind experimental realization. This deserves more thorough discussion/explanation in order to be used as a “selling point” (on which the "urgency" "significance" criteria are both hinging; and a good one but cryptic, unsubstantiated here) for a Letter. It may be buried somewhere in the text but I am running out of time… So, I have to call the bluff here or, of the authors have explained already how this all meant to work, I’d stand corrected. With this addition, possibly missed by me or understated by the authors, paper can be recommended. Minor/major revision? Minor since does not call for any factula work or computations; major because I feel it is necessary.

(I have read and mostly agree with other R1 and R2, as well as the authors responses. The title may be a bit too broad—kind of undercutting potential others’ paper on other may be stronger effect in other material; this is up to the Editors.)

Author's Response to Peer Review Comments:

11/12/2023

Dear Prof. Editor,

We thank you for communicating with us the review reports on our manuscript (jz-2023-02681c) titled “Nanotube slidetronics”, recently submitted for your consideration for publication in *The Journal of Physical Chemistry Letters*. We also want to express our sincere appreciation to the referees for providing valuable comments and suggestions that have helped us in improving our manuscript.

Attached to this resubmission, please find our point-by-point response to all the comments raised by the referees and their corresponding suggestions. Also, please find attached the revised manuscript, including the highlighted modifications.

We believe that our responses have adequately addressed the referees’ comments and we hope that you will share our view that our manuscript, in its revised form, merits publication in *The Journal of Physical Chemistry Letters*.

Sincerely yours,

Prof. Michael Urbakh

School of Chemistry,

Tel Aviv University

Tel Aviv 6997801, Israel

Reviewer 3

"In this manuscript, the authors predicted one-dimensional slidetronics in double-walled boron-nitride nanotubes, which the change of local electrostatic polarization was studied by controlling the chirality, wall diameter and wall slip distance of DWBNNT. This work was interesting. But I have several comments before recommending this work for publication in the Journal of Physical Chemistry Letters."

We thank the reviewer for the positive evaluation of our manuscript.

"1) The abstract is too brief and cannot clearly express the core content of the manuscript, which is not conducive to readers' reading."

We thank the reviewer for raising this stylistic point. We have carefully read through the abstract yet again. We find that it expresses well the core content of the paper as well as the main conclusions. If the reviewer has any specific core content in mind that the present version of the abstract fails to convey, we would be happy if s/he can share it with us so that we can augment the abstract. Otherwise, we find no reason to extend the abstract beyond what is absolutely required. As the great Prof. Einstein once said, "Everything should be made as simple as possible, but not simpler".

"And the author does not specify how parallel and anti-parallel are defined."

We thank the referee for pointing out the missing definition. In flat *h*-BN bilayer structures, parallel stacking is a stacking configuration, where the two surfaces can be superimposed just via spatial shift without any rotations. Anti-parallel stacking is obtained by laterally rotating one of two parallel stacked layers by 180°. The (anti-)parallel DWBNNT configuration is obtained by rolling-up the corresponding (anti-)parallelly stacked *h*-BN bilayer section. We have added this explanation as an endnote in the revised text.

"Meanwhile, its full name should be written when the abbreviation DWBNNT the first appears."

We thank the reviewer for identifying this problem. The abbreviation has been defined in the revised text.

"In addition, some references cannot be found, such as Ref. 31."

We fail to understand why the referee was unable to find Ref. 31. The DOI of this paper is: https://doi.org/10.1021/ct200880m

"2) The article was mentioned ferroelectricity at the beginning, but the author only discusses local electrostatic polarization of DWBNNT later. The polarization of double-layer h-BN slip without bended is reversible. Is it possible to be flipped polarize of the DWBNNT?"

Similar to the case of *h*-BN multilayers, where local polarization can be switched (flipped) via interlayer sliding, we demonstrate in the present manuscript that the local radial polarization of BNNTs can be reversibly switches via axial interwall shifts. Therefore, the answer to the reviewer's question is yes.

"3) How to realize the slip electronics of DWBNNT experimentally? Is the electrical polarity generated by this slip reliable? What are its potential applications?"

The answer to these questions raised by the reviewer appears in the conclusions section of the paper, where it is now written: "Measurement of these predicted effects requires the experimental interwall manipulation[27, 36-39] of facetted multi-walled nanotubes[24-29] and local probing of the resulting polarization variations.[4-6] Similar to the case of 2D ferroelectric layered materials,[4] such local probing could also be used to trigger domain wall shifting and induce reversible polarization switching, which could be utilized in memory devices. Furthermore, the intrinsically low inter-wall friction characteristics of multi-walled nanotubes supports the fabrication of coaxial sliding GHz oscillators[40-43]. By connecting local probes (e.g. conducting tips) to the outer wall of the oscillator, the periodic local polarization variations could generate AC currents, thus supporting the realization of nano-generators.". The reliability of the polarization switching was demonstrated for 2D material stacks (see, e.g., *Science* **372**, 1462-1466 (2021).). For 1D materials this, naturally, remains to be verified by experiments that will hopefully follow our theoretical prediction.

Reviewer: 4

"The work reports variations in hBN stacking across the body/area of two concentric BNNT's. Stacking variations in turn, as well discussed in the literature for planar hBN bilayers, a trendy topic, cause minor variation of polarization (normal component. This

is what the authors explore and map. The group is reputable and known for reliable BN-BN potentials as well as superlubricity for incommensurate situations, both relevant to this study. So overall results are nice, qualitatively intuitive, no surprises, and quantitatively can be trusted."

We thank the reviewer for the positive evaluation of our manuscript.

"One thing I am not sure what/how are those nanogenerator and memory devices will work!? What is generated? What/how is switched? How the "bits" are written in and read-put in memory case? Conceptually, in principle, never mind experimental realization. This deserves more thorough discussion/explanation in order to be used as a "selling point" (on which the "urgency" "significance" criteria are both hinging; and a good one but cryptic, unsubstantiated here) for a Letter. It may be buried somewhere in the text but I am running out of time… So, I have to call the bluff here or, of the authors have explained already how this all meant to work, I'd stand corrected. With this addition, possibly missed by me or understated by the authors, paper can be recommended. Minor/major revision? Minor since does not call for any factula work or computations; major because I feel it is necessary."

We thank the referee for raising this question, which gives us the opportunity to better convey potential applications of the predicted phenomenon. The experimental demonstration of GHz oscillator has been presented in reference[40-43]. When the oscillating system is electrically polar, such interwall oscillations will demonstrate GHz local potential variations. By connecting local probes (e.g. conducting tips) to the outer wall of the oscillator, one should be able to generate AC currents. Regarding read/write operations, such effects have been already demonstrated in earlier studies on two-dimensional ferroelectric materials, where the local surface polarization was reversibly switched using external potentials via a domain wall slidetronic mechanism (see M. Vizner Stern, Y. Waschitz, W. Cao, I. Nevo, K. Watanabe, T. Taniguchi, E. Sela, M. Urbakh, O. Hod, and M. Ben Shalom, "Interfacial Ferroelectricity by van der Waals Sliding", *Science* **372**, 1462-1466 (2021)). Similar local domain wall sliding manipulation can be envisioned for the 1D counterparts studied in the present paper.

Since the present letter focuses on the predicted phenomenon, rather than on its technological utilization, we defer explicit exploration of potential applications to future studies.

To better convey this point, we modified the outlook section of the manuscript as follows: "The rich polar domain variation physics exhibited by DWBNNTs under coaxial inter-wall sliding predicted herein constitutes the first demonstration of 1D slidetronics. By controlling the chiral indices of the two tube walls one may design a plethora of DWBNNT structures with pre-determined circumferentially facetted super structures.[29-30] These, in turn, lead to diverse slidetronic characteristics, ranging from strong local variations of the electrostatic potential energy to delocalized chiral polar

domain dynamics. Measurement of these predicted effects requires the experimental interwall manipulation[27, 36-39] of facetted multi-walled nanotubes[24-29] and local probing of the resulting polarization variations.[4-6] Similar to the case of 2D ferroelectric layered materials,[4] such local probing could also be used to trigger domain wall shifting and induce reversible polarization switching, which could be utilized in memory devices. Furthermore, the intrinsically low inter-wall friction characteristics of multi-walled nanotubes supports the fabrication of coaxial sliding GHz oscillators.[40-43] By connecting local probes (e.g. conducting tips) to the outer wall of the oscillator, the periodic local polarization variations could generate AC currents, thus supporting the realization of nano-generators.

"(I have read and mostly agree with other R1 and R2, as well as the authors responses. The title may be a bit too broad—kind of undercutting potential others' paper on other may be stronger effect in other material; this is up to the Editors.)"

As far as we know, this is the first time that a one-dimensional slidetronic effect is predicted. Since the discovered phenomenon, demonstrated herein for BNNTs, is of general nature, we strongly believe that a broader title is adequate. Hence, we opt to keep the original title, which clearly conveys the message of a newly predicted 1D slidetronic phenomenon.
